# LOOKING UPSTREAM: THE ASSOCIATION BETWEEN AFFORDABLE HOUSING, NURSING HOME USE, AND UNMET CARE NEEDS IN THE COMMUNITY

**DOI:** 10.1093/geroni/igz038.558

**Published:** 2019-11-08

**Authors:** Meghan Jenkins Morales, Stephanie Robert

**Affiliations:** University of Wisconsin-Madison, Madison, Wisconsin, United States

## Abstract

In the U.S., population aging is coinciding with a growing affordable housing crisis. Evidence suggests that housing security contributes to health, but less is known about how affordable housing affects aging in place. We use a nationally representative sample (n=5,117) of older community-dwelling Medicare beneficiaries from the 2015 National Health and Aging Trends Study to test the association between housing cost burden (HCB) and moving to a nursing home, death, or remaining in the community by 2017. Among 2017 community-stayers (n=4,836), we also test the association between HCB and unmet care need, defined as experiencing a consequence related to 12 mobility (e.g., stayed in bed), self-care (e.g., skipped meals) and household (e.g., no clean laundry) activities. HCB is the proportion of income spent on rent or mortgage: low (<30%), moderate (30-50%), severe (≥50%), or home paid off (referent). Among nursing home movers, 26% had moderate or severe HCB in 2015 compared to 16% of community-stayers. Informed by the person-environment fit perspective, weighted stepwise regression models (multinomial and logistic) adjust for race, age, sex (Model 1), self-rated health, probable dementia (Model 2), living with others and high income (Model 3). Severe HCB is significantly associated with nursing home entry (RRR=2.66, SE=0.89) and this association is only partially mediated by health factors (RRR=2.16, SE=0.72) and resources (RRR=1.95, SE=0.64). Among community-stayers, severe HCB is significantly associated with unmet care need across all models. This study suggests that affordable housing is an important protective factor for older adults to age well in the community.

